# Hip Range of Motion and Strength in Male Athletes with Stage 1 Osteitis Pubis: A Cross-Sectional and Correlational Study

**DOI:** 10.3390/ijerph191912824

**Published:** 2022-10-06

**Authors:** Luis Ceballos-Laita, Ignacio Hernando-Garijo, Ricardo Medrano-de-la-Fuente, María Teresa Mingo-Gómez, Andoni Carrasco-Uribarren, Sandra Jiménez-del-Barrio

**Affiliations:** 1 Clinical Research in Health Sciences Group, Department of Surgery, Ophthalmology and Physiotherapy, University of Valladolid, 42004 Soria, Spain; 2 Physiotherapy Department, International University of Catalunya, 08195 Barcelona, Spain

**Keywords:** osteitis pubis, range of motion, strength, pain, athletes

## Abstract

Background: The objectives of this study were: (1) to determine whether athletes with stage 1 osteitis pubis (OP) present differences in hip range of motion (ROM) and muscle strength, between both sides and compared with healthy athletes; (2) to investigate the relationship between the internal rotation (IR) ROM and pain intensity and physical function. Methods: a cross-sectional and correlational study was designed, in which 30 athletes (15 athletes with stage 1 OP and 15 healthy athletes) were included. Pain intensity, physical function, hip ROM and hip muscle strength were assessed. Results: The ROM assessment reported significant differences between both groups in the IR, external rotation (ER) and adduction (ADD) ROM of the painful side (PS) (*p* < 0.05). The OP group showed differences between both sides in IR ER and ADD ROM (*p* < 0.05). No statistically significant differences were found between or within groups in the maximum isometric strength of the hip (*p* > 0.05). A strong negative correlation between pain intensity and IR ROM (r = −0.640) and a strong positive correlation between physical function and IR ROM (r = 0.563) were found in the OP group. Conclusions: Male athletes with stage 1 OP present a hip IR, ER and ADD ROM limitation in the PS compared to non-PS and to healthy athletes. IR ROM is correlated to pain intensity and physical function in athletes with stage 1 OP.

## 1. Introduction

Athletic osteitis pubis (OP) or pubic bone stress injury is a chronic, painful overuse pathology of the pubic symphysis and the adjacent para-symphyseal bone suffered by athletes [1]. It is characterized by unilateral or bilateral groin and/or pubic pain. The pain may radiate to the symphysis, adductor, abdominal, perineal, inguinal and/or scrotum regions and is exacerbated by running, kicking, turning, twisting, cutting, pivoting, or sprinting, causing physical function limitations in daily living and sport activities [2,3].

The prevalence of OP has shown to be between 0.5% and 14% [4] but is not well determined because of the lack of epidemiological studies [5,6] and the different terms used in the literature such as sport hernia, athletic pubalgia, groin disruption injury or “Gilmore’s” groin, among others [2,3]. Using diverse terms makes it difficult to establish a clear prevalence and a clear comprehension of this pathology.

OP diagnosis is mainly based on magnetic resonance imaging (MRI) and physical examination. MRI is currently considered the gold standard for OP diagnosis and is also used to confirm the diagnosis and/or to exclude other pathologies. The most common findings in MRI are bone marrow edema, osseous irregularity, bone resorption, periosteal reaction, osteophytes, fatty degeneration, subchondral cyst, tendon lesions and fluid in the symphysis [7,8]. The physical examination should include three provocation tests: squeeze test, single adductor test and bilateral adduction test [9] and is crucial to classify the clinical stage.

The OP can be classified in four clinical stages based on the physical examination. This classification explores the side, the site and the characteristics of pain: (Stage 1) Unilateral inguinal pain with radiation to adductors. In this stage, the pain disappears after warm-up and worsens after training. (Stage 2) bilateral inguinal and adductor pain that is exacerbated after training. (Stage 3) Bilateral pain in the groin, adductor, suprapubic and/or abdominal regions. In this stage, the pain appears during training, kicking, sprinting, turning, etc. (Stage 4) Generalized pain radiated to lumbar region that appears during daily living activities [10,11].

During the physical examination, clinicians pay attention to regions adjacent to the pubic symphysis, such as the hip joint. Some authors have discussed that hip range of motion (ROM) limitations [12,13,14] or hip muscle impairments may have a direct influence on the pelvic bone [15]. A cadaveric study concluded that the decrease in the physiological internal rotation (IR) ROM of the hip in activities that require more functional rotation, can increase the stress in the pelvic bone as a compensatory mechanism [13]. Repetitive loading of the pubic symphysis could cause symphyseal hypermobility or instability, which has been associated with para-symphyseal tendon injury, rectus abdominis and adductor longus tears and OP [16,17].

Most of the studies have assessed the IR ROM in patients with cam-type or pincer-type femoro-acetabular impingement (FAI) and OP [13,18,19,20]. Only two studies have evaluated the isokinetic imbalance of the hip muscles in athletes with OP [21,22]. However, to the best of our knowledge, no study has analysed the hip ROM and hip muscle strength in athletes with stage 1 OP and without any intra-articular hip pathology. Thus, the objectives of this study were: (1) to determine whether athletes with stage 1 OP present differences in hip ROM and hip muscle strength in the painful side (PS) compared to the non-PS and to healthy athletes; (2) to investigate the relationship between the IR ROM, pain intensity and physical function in athletes with stage 1 OP.

## 2. Materials and Methods

### 2.1. Study Design

A cross-sectional study was carried out between January and June 2022. This study was approved by the Research Ethics Committee of Valladolid Este (PI-22-2612) and followed the Strengthening the Reporting of Observational Studies in Epidemiology (STROBE) guideline [23]. The study was performed according to the Declaration of Helsinki (2013) and the Taipei criteria (2016). All the participants were informed about the objective of the study, agreed to participate and signed the informed consent form.

### 2.2. Participants

Thirty male volunteers participated in the study (fifteen athletes diagnosed with stage 1 OP and fifteen healthy athletes). The inclusion criteria in the OP group were based on the Giai Via et al. [10] recommendations: (1) athletes with unilateral groin and/or pubic pain for more than 3 months; (2) the pain had to disappear after warm-up and worsen after training; (3) squeeze test, single adductor test and bilateral adductor test had to provoke symptoms in the patients; (4) MRI had to present OP findings evaluated by a radiologist [10]. The inclusion criteria for the control group were: (1) athletes without pain in the groin/or pubic region for more than 3 months. Exclusion criteria for both groups were: (1) hip pain due to an intra-articular pathology (FAI, acetabular labral tears, chondral lesions, osteoarthritis, osteonecrosis, dysplasia, or fractures); (2) hip pain due to an inflammatory disease; (3) previous hip, pelvis or lumbar spine surgery or fracture; (4) unclear results in the clinical test or in the MRI.

### 2.3. Procedure

Before participants’ enrolment, the MRIs were evaluated for OP findings. Then, the athletes underwent a physical examination in which the squeeze test, single adductor test and bilateral adduction test were applied. Athletes were included if OP findings were found in the MRI and if the pain of the patients was reproduced by the clinical tests.

Sociodemographic, sport-related variables, pain intensity, pain localization and physical function were measured for descriptive purposes. The clinical variables were hip abductor, adductor, IR and external rotation (ER) ROM, and hip abductor, adductor, IR and ER muscle strength. All variables were assessed in the PS and non-PS by two examiners. The examiners were blinded to the group allocation and to the PS of each subject. The lower limb assessment was selected randomly.

### 2.4. Reliability of the Measures

Test–retest reliability was assessed for all the variables before the study. The measurements were performed by a professional physiotherapist with more than ten years of clinical experience. Ten healthy athletes, different from the study participants, were assessed on the same day with 10 min between evaluations. Hip ROM and hip muscle strength were assessed similarly to that conducted in this study. These measures were used to calculate the intraclass correlation coefficient (ICC) (Table 1).

### 2.5. Pain Intensity and Localization

Pain intensity was recorded using the Numeric Rating Scale (NPRS) in which 0 points represented no symptoms and 10 points represented the most intense pain imaginable. The NPRS has shown an excellent test–retest reliability (ICC: 0.95) [24].

Pain localization was measured using a body chart. Athletes could describe the location of pain with more than one descriptor. Locations were defined as follows: (1) groin: area between the inguinal ligament and proximal part of the femur; (2) pubic: symphysis pubic area; (3) adductor: area between the bottom part of the symphysis pubis and the medial part of the tight.

### 2.6. Physical Function

The Copenhagen Hip and Groin Outcome Score (HAGOS) is a valid questionnaire designed to assess hip and groin pain and function. It is composed of six separate subscales: Pain, Symptoms, Physical Function in Daily Living, Physical Function in Sports and Recreation, and Hip- and Groin-related Quality of Life. The HAGOS questionnaire ranges from 0 to 100, where 0 indicates extreme groin pain and limitations while 100 infers freedom from, symptoms and full function. The test–retest reliability has shown an ICC ranging from 0.82 to 0.91 for the six subscales [25].

### 2.7. Range of Motion

Hip ROM was measured using an inclinometer and a universal goniometer according to the procedure described by Pua et al. [26]. The test–retest reliability of this protocol has shown to be excellent for IR, ER, ABD and ADD ROM (ICC: 0.89–0.97).

### 2.8. Isometric Strength

Hip muscles strength was measured using a Hand-held dynamometer (Lafayette 01165). Hip abductor and adductor muscles were measured according to the protocol described by Mentiplay et al. [27]. The test–retest reliability of this protocol has been shown to be excellent (ICC: 0.84–0.91). Hip internal and external rotator muscles were recorded following the protocol described by Pua et al. [26]. The test–retest reliability of this protocol has also been shown to be excellent (ICC: 0.98). The hand-held dynamometer was configured to record the maximal force of the hip muscles in Newtons. All participants performed two trials. Each trial lasted from 3 to 5 s and a rest interval of 1 min was established between each trial. In each trial, the participants were encouraged to push as hard as possible. The value recorded in each trial was the maximum isometric force and the mean of both tests was considered for statistical purposes.

### 2.9. Statistical Analysis

G*Power 3.1. (Universität Düsseldorf, Düsseldorf, Germany) was used for calculating the statistical power with a 95% confidence interval and a sample of 30 participants. The estimation provides 99% power for IR and ADD ROM and 60% power for ER ROM differences between groups The statistical power obtained for correlation was 99% for IR ROM and pain intensity correlation and 97% power for physical function and IR ROM correlation.

SPSS version 20.0 (IBM Corporation, Armonk, NY, USA) for Windows was used for statistical analysis. The test–retest reliability was calculated for all the variables. The reliability was considered excellent when the values of ICC exceeded 0.75. When the ICC ranged from 0.4 to 0.74, the reliability was considered good to fair and when the value was less than 0.4 it was considered poor [28].

Quantitative variables were presented as Mean (M) and standard deviation (SD). The Shapiro Wilk-test was used to evaluate the normal or non-normal distribution of the variables. Between-group comparisons of clinical and demographic variables were analysed using the Student’s *t*-test or the Mann-Whitney U test, for normally distributed data or non-normally distributed data. Between hips comparisons were analysed using the paired t-test or the Wilcoxon test, for normally distributed data or non-normally distributed data. To investigate the correlation between pain intensity, physical function and IR ROM, Spearman Rho was used due to the small number of participants. A *p*-value < 0.05 was considered statistically significant. The Rank Correlation Coefficients were interpreted as weak (rho = 0–0.3), moderate (rho = 0.3–0.5). strong (rho = 0.5–0.7) or very strong (rho = 0.7–1) [29].

## 3. Results

Fifty athletes were recruited for the study. Twenty were excluded for not meeting the eligibility criteria. Finally, thirty participants (fifteen athletes diagnosed with stage 1 OP and fifteen healthy matched controls) were included in the study. Sociodemographic and sport-related variables were similar at baseline without statistically significant differences between them (Table 2).

**Figure 1:**
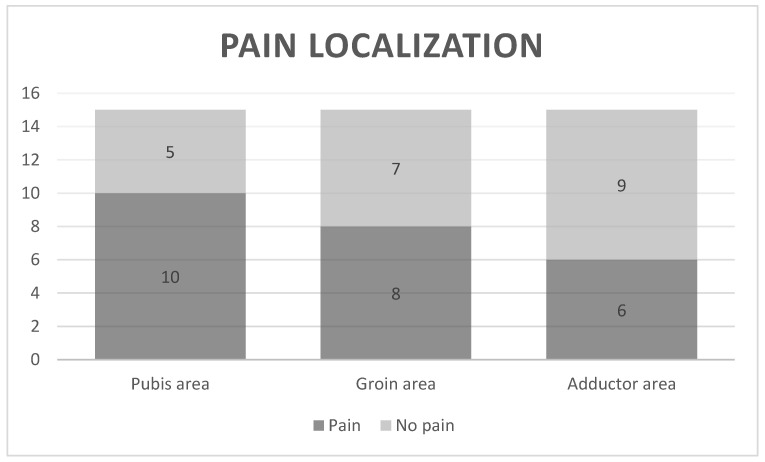
Pain localization in the body chart.

Pain intensity, pain localization and physical function assessment showed that the OP group presented a pain intensity of 6.75 ± 1.49 points. The body chart showed that 66.66% of the participants in the OP group presented pain in the pubis area (n = 10), 53.33% (n = 8) in the groin area and 40% (n = 6) in the adductor area (Figure 1). The physical function measured with the HAGOS questionnaire showed a mean value of 54.20 ± 20.33.

The ROM assessment reported statistically significant differences between both groups in the IR ROM (Δ-11.30; 95%CI: −15.58, −7.01; *p* < 0.001), ER ROM (Δ-5.70; 95%CI −11.24,−0.15; *p* = 0.044) and ADD ROM (Δ-5.63; 95%CI: −7.05, −4.21; *p* < 0.001), of the PS and in the IR ROM (Δ-7.25; 95%CI −13.73, −0.76; *p* = 0.014) of the non-PS. Within-groups comparisons showed statistically significant differences between the PS and the non-PS in IR ROM (Δ-4.45; 95%CI −6.93, −1.96; *p* = 0.003), ER ROM (Δ-7.20; 95%CI −11.67, −2.72; *p* = 0.005) and ADD ROM (Δ-3.70; 95%CI −4.71, −2.68; *p* < 0.001), in the OP group. No within-group differences were found in the control group (*p* > 0.05) (Table 3).

No statistically significant differences were found between or within groups in the maximum isometric strength of the hip (*p* > 0.05). Only in the within group analysis were differences for ER strength in the OP group found (Δ-1.24; 95%CI −1.96, −0.52; *p* = 0.004) (Table 4).

**Figure 2:**
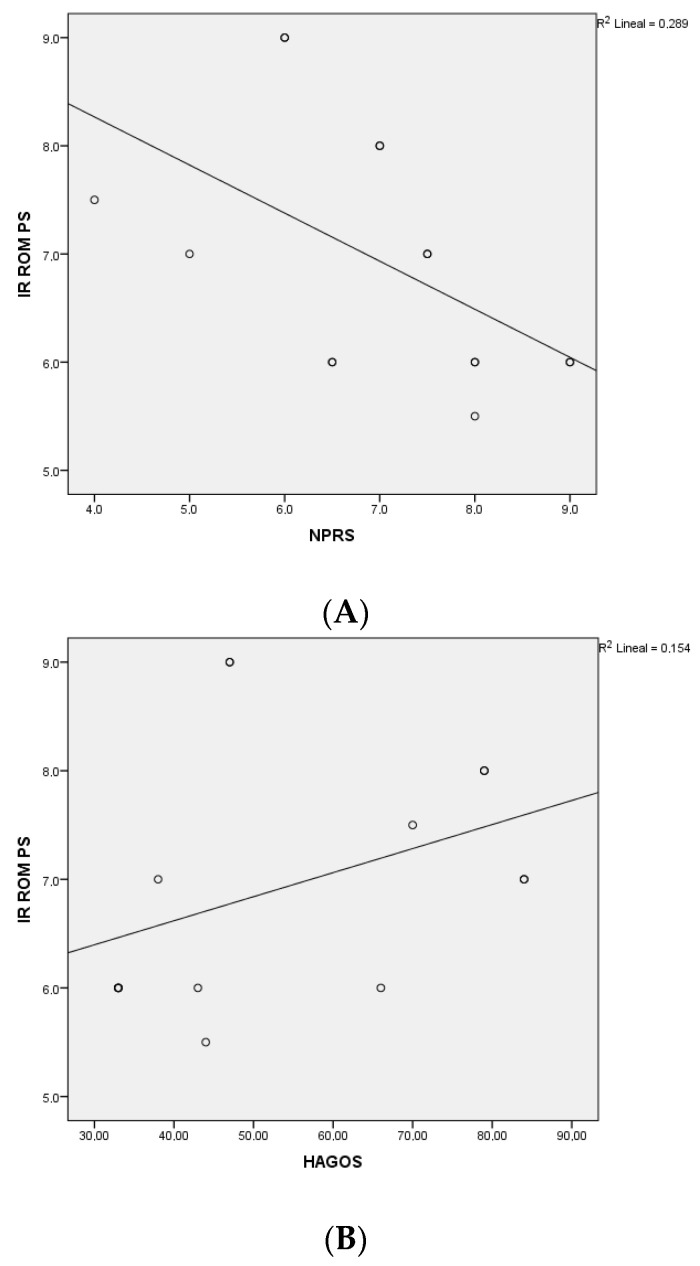
(**A**). Correlation analysis between IR ROM and pain intensity. (**B**). Correlation analysis between IR ROM and physical function.

The correlation analysis showed a strong negative correlation between pain intensity and IR ROM (r= −0.640; *p* = 0.010) (Figure 2A) and a strong positive correlation between physical function and IR ROM (r= 0.563; *p* = 0.029) in the OP group (Figure 2B).

## 4. Discussion

The objectives of this study were to determine if athletes with stage 1 OP presented differences in hip ROM and hip muscle strength in the PS, compared to non-PS and to healthy athletes, and to investigate the relationship between IR ROM, pain intensity and physical function. The results of this study confirmed the hypothesis that athletes with stage 1 OP included in the study presented an IR, ER and ADD ROM limitation in the PS compared to the non-PS and to healthy athletes. In addition, the IR ROM was negatively correlated to pain intensity and physical function.

The athletes included in this study presented unilateral pain, mainly in the pubic and groin area, that radiated to adductors in some cases. The pain decreased after warm-up and got worse after training, causing some physical function limitations. These clinical characteristics evidence the athlete’s classification as stage 1 [10]. In addition, the MRI showed OP findings but no hip intra-articular injuries. The population of this study met the inclusion criteria described by Giai Via et al. [10]. However, the studies carried out in athletes with OP have not described the clinical characteristics of the patients according to the clinical stages of OP [9,14] or have not assessed the presence of hip intra-articular pathologies [21,22].

The results of our study showed that patients with stage 1 OP presented IR, ER and ADD ROM limitations. These results are in accordance with previous studies that found a hip rotation deficit in patients with OP [9,12,14]. However, this is the first study that found a hip IR, ER and ADD ROM limitation in patients with stage 1 OP but without FAI. This suggests that not only hip intra-articular pathologies but other structures such as soft tissues could produce the ROM restriction.

Most studies to date have focused on the role of IR ROM in OP [13,18,19,20]. The functional IR ROM for athletes has been reported to be around 30° [30]. The mean IR ROM value of our study was 6.80ª [1,11]. Birmingham et al. [13] demonstrated that the IR ROM limitation cause an increment of the symphysis pubic motion. The repetitive loading of the pubic symphysis causes a hypermobility which has been associated with OP [16]. However, the results of this study suggest that not only IR ROM should be measured in OP, but also ER and ADD ROM should be taken in consideration. The reduction in three-dimensional movement of the hip may be compensated for by the increase of the symphysis pubis movement.

The reduction of the IR ROM has been established as one of the most important risk factors for the development of groin or pubic pain [12,31]. Few authors have clinically described this characteristic in patients with groin and/or pubic pain [9,32]. In addition, Birmingham et al. [13] explored in a cadaveric study the role of the IR ROM on the pubic symphysis motion, but no study has assessed the relationship between the IR ROM and the pain intensity and physical function in patients with OP. The results of our study found a strong negative correlation between IR ROM and pain intensity and a strong positive correlation between IR ROM and physical function, which means that the greater the hip IR ROM reduction, the greater the pain intensity and the physical function limitations. This suggests that the IR ROM plays an important role in athletes with OP.

No differences were found for hip muscle isometric strength between both legs or between both groups. Our study found similar results to those of Mohammad et al. [21], who studied the isokinetic strength of hip abductors and adductors and found no differences comparing the PS to the non-PS and to a control group, though a significantly higher time to peak torque, acceleration and deceleration times were found. The results of our study could be influenced by the measurement tool. The maximum isometric strength was evaluated using a hand-held dynamometer but this may not be the best tool for athletes. The assessment of the dynamic strength with a different instrument may reflect other results, especially in the hip IR and ER strength.

The present study has several limitations. First, only male athletes with stage 1 OP or male healthy athletes were included, so the results cannot be extrapolated to other populations. Second, the number of athletes included in each group is limited and may not be sufficiently representative of the population, Third, we did not assess the dynamic muscle strength in all the three planes of the hip, which could show other significant results. Finally, the cross-sectional design does not allow associating cause–effect of the differences achieved.

Future studies should investigate other genders and other athletes in different OP clinical stages. In addition, a methodological design is necessary that allows subjects to be followed over time to establish casual associations.

## 5. Conclusions

Male athletes with stage 1 OP present a hip IR, ER and ADD ROM limitation in the PS compared to the non-PS and to healthy athletes. IR ROM is correlated to pain intensity and physical function in athletes with stage 1 OP.

## Figures and Tables

**Table 1:** Test–retest reliability of the study variables.

Outcome	ICC (95% CI)
ABD ROM (°)	0.97 (0.96,0.98)
ADD ROM (°)	0.98 (0.97,0.99)
ER ROM (°)	0.92 (0.9, 0.94)
IR ROM (°)	0.98 (0.96,0.99)
ABD Strength (kg)	0.97 (0.95,0.98)
ADD Strength (kg)	0.95 (0.9,0.97)
ER Strength (kg)	0.98 (0.97,0.99)
IR Strength (kg)	0.97 (0.94,0.98)

ICC: Intraclass correlation coefficient; CI: Confidence interval; ABD: Abduction; ADD: Adduction; ER: External rotation; IR: Internal rotation; ROM: Range of motion.

**Table 2:** Demographic and sport-related variables.

	OP Group M (SD)	Control Group M (SD)	*p*-Value
Age (years)	25.30 (4.42)	22.73 (2.68)	0.123
Weight (kg)	77.68 (8.79)	73.86 (8.62)	0.294
Height (cm)	181.60 (8.15)	178.33 (6.92)	0.293
BMI (kg/cm^2^)	23.46 (1.45)	23.15 (1.98)	0.680
Weekly practice (hours)	10.01 (4.34)	10.52 (5.66)	0.809
Frequency (days/week)	4.20 (0.81)	3.80 (1.27)	0.637

OP: Osteitis pubis; M: Mean; SD: Standard Deviation; BMI: Body mass index.

**Table 3:** Analysis of hip ROM.

	OP Group (n = 15)		Control Group (n = 15)		
Outcomes		Differences (*p*-Value)		Differences (*p*-Value)	Between-Groups Differences *p*-Value
IR ROM (°)					
PS	6.80 (1.11)	−4.45 (−6.93; −1.96) 0.003	18.10 (6.43)	−0.40 (−4.13; 3.33) 0.822	−11.30 (−15.58; −7.01) <0.001
Non-PS	11.25 (3.51)		18.50 (9.43)		−7.25 (−13.73; −0.76) 0.014
ER ROM (°)					
PS	16.40 (5.58)	−7.20 (−11.67; −2.72) 0.005	22.10 (7.79)	−0.86 (−3.46; 1.73) 0.486	−5.70 (−11.24; −0.15) 0.044
Non-PS	23.60 (8.84)		22.96 (1.52)		0.63 (−6.27; 7.54) 0.851
ABD ROM (°)					
PS	9.80 (4.51)	−1.20 (−3.87; 1.47) 0.336	11.47 (2.92)	0.66 (−3.15; 4.48) 0.714	−1.67 (−4.73; 1.40) 0.273
Non-PS	11.00 (7.36)		10.80 (6.71)		0.20 (−5.68; 6.08) 0.945
ADD ROM (°)					
PS	0.30 (0.48)	−3.70 (−4.71; −2.68) < 0.001	5.93 (2.12)	0.13 (−1.72; 2.03) 0.883	−5.63 (−7.05; −4.21) < 0.001
Non-PS	4.00 (1.05)		5.80 (3.46)		−1.80 (−4.15; 0.55) 0.076

OP: Osteitis pubis; ABD: Abduction; ADD: Adduction; IR: Internal rotation; ER: External rotation; ROM: Range of motion; PS: Painful side.

**Table 4:** Analysis of hip strength.

	OP Group (n = 15)		Control Group (n = 15)		
Outcomes		Differences (*p*-Value)		Differences (*p*-Value)	Between-Groups Differences *p*-Value
IR Strength (kg)					
PS	7.58 (2.97)	0.93 (−1.79; 3.66) 0.458	6.96 (1.52)	0.44 (−0.12; 1.00) 0.116	0.61 (−1.25; 2.47) 0.503
Non-PS	6.64 (1.78)		6.52 (1.41)		0.11 (−1.20;1.44) 0.855
ER Strength (kg)					
PS	7.23 (2.28)	−1.24 (−1.96; −0.52) 0.004	7.79 (2.59)	0.26 (−0.61; 1.13) 0.533	−0.56 (−2.65; 1.52) 0.584
Non-PS	8.48 (2.89)		7.53 (1.82)		0.94 (−1.00; 2.88) 0.326
ABD Strength (kg)					
PS	10.01 (3.41)	0.07 (−0,86; 1.00) 0.869	10.80 (3.99)	0.22 (−0.76; 1.20) 0.638	−0.79 (−3.98; 2.39) 0.610
Non-PS	9.94(1.59)		10.58 (3.05)		−0.64 (−3.29; 2.00) 0.618
ADD Strength (kg)					
PS	9.01 (4.15)	1.18 (−2.33; 4.70) 0.466	9.82 (2.47)	−0.02 (−0.72; 0.67) 0.936	−0.81 (−3.54; 1.92) 0.545
Non-PS	7.83 (2.05)		9.85 (2.75)		−2–20 (−4.13; 0.09) 0.060

OP: Osteitis pubis; ABD: Abduction; ADD: Adduction; IR: Internal rotation; ER: External rotation; PS: Painful side.

## Data Availability

The anonymized data are available from the author upon reasonable request.
